# Supplementary material for: Nanotopography Evaluation of NiTi Alloy Exposed to Artificial Saliva and Different Mouthwashes
Source: Materials (Basel). 2022 Dec 6;15(23):8705. doi: 10.3390/ma15238705 (PMC9739356; doi:10.3390/ma15238705)
Supplement: Supplementary file 1 [file materials-15-08705-s001.zip › materials-2022672-supplementary.pdf]

materials

Supplementary Materials

Nanotopography Evaluation of NiTi Alloy Exposed to Artificial Saliva and Different Mouthwashes

Zoran Bobić *, Sanja Kojić, Goran M. Stojanović, Vladimir Terek, Lazar Kovačević and Pal Terek

Faculty of Technical Sciences, University of Novi Sad, Trg Dositeja Obradovića 6, Novi Sad, 21000, Serbia
* Correspondence: zoranbobic@uns.ac.rs; Tel.: +381-21-485-2330

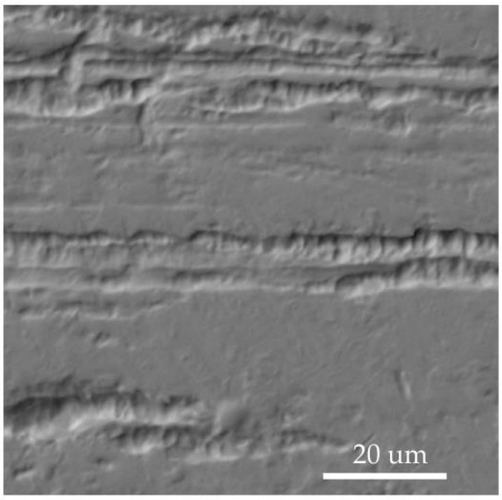

(a)

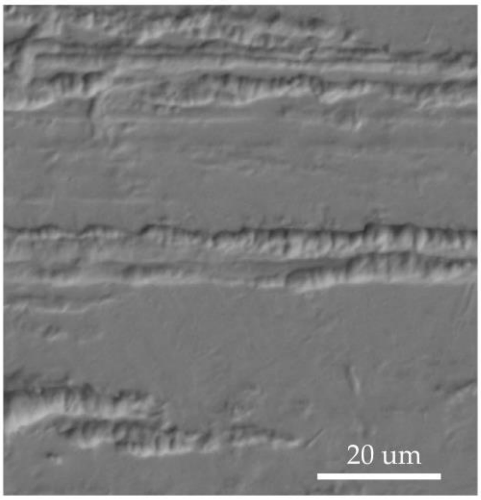

(b)

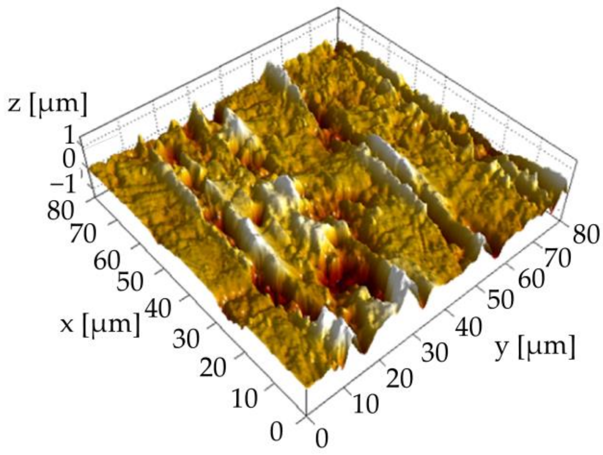

(c)

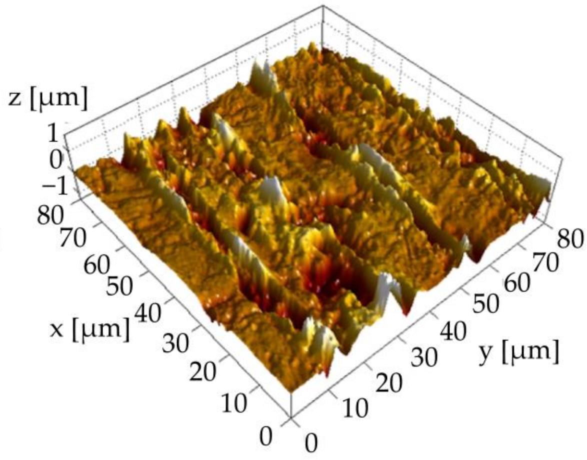

(d)

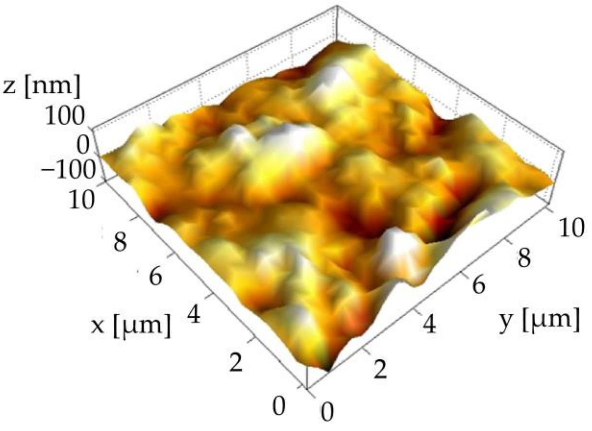

(e)

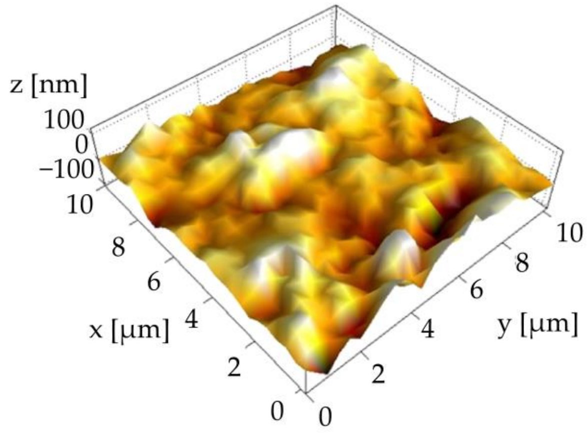

(f)

Figure S1. Representative images before (left) and after the corrosion test (right). Artificial saliva (Sample 1): (a,b) SEM images of 80 × 80 μm area; (c,d) AFM topography images of the same 80 × 80 μm area; (e,f) AFM topography images of cropped 10 × 10 μm smooth area between the grooves.

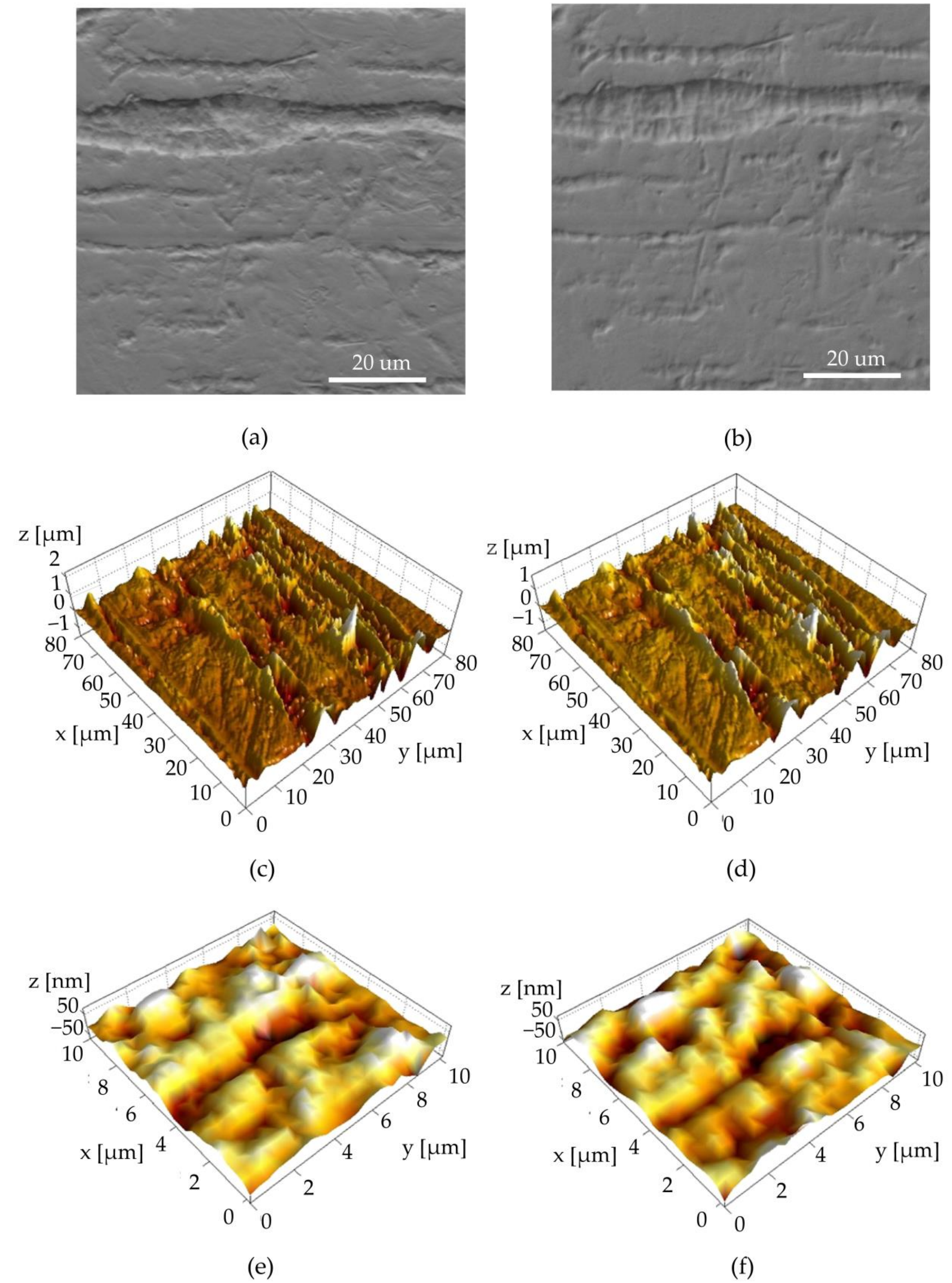

Figure S2. Representative images before (left) and after the corrosion test (right). Aquafresh Big teeth® (Sample 2): (a,b) SEM images of 80 × 80 µm area; (c,d) AFM topography images of the same 80 × 80 µm area; (e,f) AFM topography images of cropped 10 × 10 µm smooth area between the grooves.

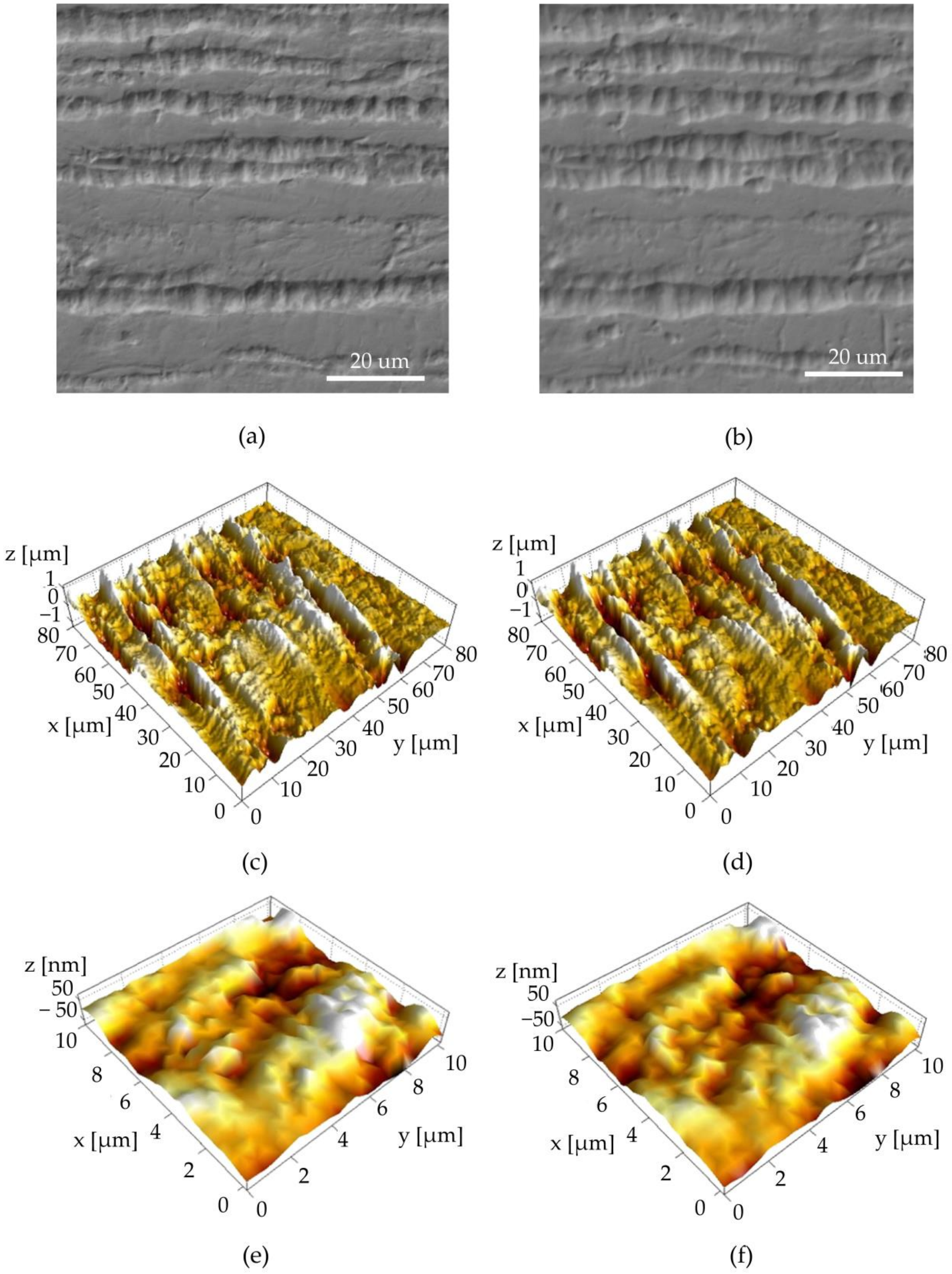

Figure S3. Representative images before (left) and after the corrosion test (right). Listerine® (Sample 4): (a,b) SEM images of 80 × 80 μm area; (c,d) AFM topography images of the same 80 × 80 μm area; (e,f) AFM topography images of cropped 10 × 10 μm smooth area between the grooves.
